# Evaluation of the Convergence Region of an Automated Registration Method for 3D Laser Scanner Point Clouds

**DOI:** 10.3390/s90100355

**Published:** 2009-01-08

**Authors:** Kwang-Ho Bae

**Affiliations:** Department of Spatial Sciences, Curtin University of Technology, GPO Box U1987, Perth, WA 6845, Australia; E-Mail: K.H.Bae@curtin.edu.au; Tel.: +61-8-9266-7604; Fax: +61-8-9266-2703

**Keywords:** Convergence region, registration, terrestrial laser scanners, point clouds

## Abstract

Using three dimensional point clouds from both simulated and real datasets from close and terrestrial laser scanners, the rotational and translational convergence regions of Geometric Primitive Iterative Closest Points (GP-ICP) are empirically evaluated. The results demonstrate the GP-ICP has a larger rotational convergence region than the existing methods, e.g., the Iterative Closest Point (ICP).

## 1. Introduction

Laser scanners provide a three-dimensional (3D) sampled representation of the surfaces of objects with high spatial resolution and have been gained popularity in terrestrial and airborne applications such as 3D-reconstruction of terrain [17], tree height estimation [36] and building segmentation [27]. Those datasets are generally called 3D point clouds. Since laser scanners have a limited field of view, it is necessary to collect data from several locations in order to obtain a completed representation of an object. These data must be transformed into a common coordinate system for further analysis. This procedure is called the registration of point clouds. A method for the automated registration of two point clouds is summarised as follows:

- Finding correct correspondence from either selected point-to-point or point-to-surface pairs.
- Adjustment algorithms for estimating the relative transformation parameters between point clouds using point- or plane-based methods, e.g. Umeyama [38], Horn [24], Haralick *et al.* [20] and so on.

A considerable amount of work on the automated registration of 3D point clouds has been conducted over the last few decades by researchers from different fields such as photogrammetry, computer vision and artificial intelligence. One can find reviews on existing registration methods from e.g. Haralick *et al.* [20], Rusinkiewicz and Levoy [32], Campbell and Flynn [9], Rodrigues *et al.* [31] and Gruen and Akca [19]. Existing automated registration methods, e.g. the Iterative Closest Point (ICP) method and its variants [7, 10], work well if good a priori alignment is provided. Chen and Medioni's [10] method is popular since its results are much precise than that of the ICP [32]. In order to minimise the search space for correspondence between two point clouds and to increase the accuracy in the selection of the corresponding points, several researchers have used geometric features, e.g. Higuchi *et al.* [21], Chua and Jarvis [11], Johnson and Hebert [25], Rabbani *et al.* [30], Brenner *et al.* [8] and Barnea and Filin [6]. Higuchi *et al.* [21] proposes a spherical map of curvature with mesh, the Spherical Attribute Images (SAI), which is similar to the Extended Gaussian Images [14, 23]. Two SAIs from two point clouds are registered to estimate the rotation angles between range imageries. Johnson and Hebert [25] use a 2D histogram of local distance and angles to neighbourhood points to recover the correspondence. Sharp *et al.* [34] proposes to use either spherical harmonics or the second order momentum to minimise the error to find the correspondence of 3D range camera datasets.

Convergence region of a registration method is defined as the size of a basin within which the registration method can find a solution close to the truth [16, 31-32]. Since the relative transformation parameters are estimated by a registration method, the convergence basin is expressed in the transformation parameter space, i.e. three rotational and three translational parameters. It is difficult to determine the convergence of a registration algorithm since it depends on both initial rotation and translation parameters. To obtain ±50 rotational convergence region around an axis in one situation does not guarantee that the same or a larger rotational convergence region will be achieved in other situations. The convergence region of Chen and Medioni's [10] method is known to be much smaller than that of the ICP. From the author's experience with their method for terrestrial laser scanner datasets, Chen and Medioni's [10] method needs about a millimeter translational error and approximately a degree of rotational error to successfully find a solution. Bae [1] and Bae and Lichti [3] proposed a pair-wise and feature-based registration method for three-dimensional point clouds named Geometric Primitive Iterative Closest Points (GP-ICP). It utilises the estimated local surface normal vectors and curvatures for geometric attributes to select the possible corresponding points. In terms of the sampling strategy of the GP-ICP, high curvature points are only used in the early iteration and gradually more points are included in the selection pool for correspondence.

Although these feature-based ICP methods [11, 14, 21, 25, 34] increase the accuracy in selecting corresponding points and the efficiency of the algorithm, a registration method with large convergence region is still to be developed [29, 31]. In this paper, an automated registration method, the GP-ICP, is introduced. Secondly, the rotation and translational convergence region of the GP-ICP are empirically investigated with simulated and real 3D point clouds from both close-range and terrestrial laser scanners.

The remaining sections of this paper are orgarnised as follows: In Section 2, the GP-ICP will be introduced and briefly discussed. In Section 3, experiments with both simulated data and close and terrestrial laser scanner datasets will be discussed.

## 2. Automated registration method for 3D point clouds

Geometric primitives, such as surface normal vector, curvature, and the change of curvature and so on, may provide additional and useful information to recover the correspondence of two point clouds. A method to find the correspondence of two point clouds using geometric primitives and a local search algorithm, named Geometric Primitive ICP (GP-ICP), is proposed. Since this paper aims to present the evaluation results of the convergence region of the GP-ICP, the precision and accuracy of the relative transformation between point clouds are treated in the paper. One can find an advanced version of GP-ICP in Bae [7], in which an outlier method using a positional uncertainty model of laser scanners [1, 4] was implemented in order to the precise estimation of the relative transformation parameters between the point clouds.

### 2.1. Metrics for finding correspondence between two point clouds

Although the simplest method of estimating the surface normal vector is the first order three-dimensional plane fitting [33], the covariance matrix will be utilised in this paper since the first order plane fitting is equivalent to the eigenvalue problem of the covariance matrix. In addition, the covariance analysis provides additional geometric information such as curvature and its higher order derivatives. Let *p*_i_ be the coordinates of *i*th point in a point cloud and note that a bold letter represents a matrix or a vector. The covariance of a point and its *k* neighbour points is expressed as:

(1) $$\text{COV}(p_{\text{i}})=\frac{1}{k}\sum_{m=1}^{k}{\text{r}}_{m}{\text{r}}_{m}^{T}=\sum_{l=0}^{2}\lambda_{l}{\text{e}}_{l}{\text{e}}_{l}^{T}$$

where **r**_m_ = **p**_i_ − **p**_centorid_, **p**_centroid_, **p**_centroid_ is the centroid of the *k* neighbourhood and **e***_l_* is the eigenvector of the (*l*+1)th smallest eigenvalue. Since **COV**(*p*_i_) is a real, positive and semi-definite matrix, its eigenvalue are always greater than or equal to zero [18]. The eigenvector of the minimum eigenvalue is the estimated normal vector of the surface formed by *p*_i_ and its neighbourhood. The other eigenvectors are the tangential vectors of the surface and if the minimum eigenvalues are close to zero, and then the surface consisting of a point and its neighbourhood is geometrically flat. If all eigenvalues are similar, then the surface is a round-shape and locally well distributed. One can find details of other methods based on the covariance analysis for 3D point clouds in [37].

There are many ways to define geometric curvature, e.g. through Gaussian and mean curvatures or using the eigenvalues of the covariance matrix [15]. It is preferable to estimate curvature directly by using points without any pre-process such as triangulation and surface fitting since it is faster to use the neighbourhood of a point than to utilise the connectivity information provided by triangulation. Hoppe *et al.* [22] proposed a covariance analysis method for the estimation of the normal vector with consistent orientation. The covariance analysis method has been also utilised for the estimation of local curvature estimation using the ratio between the minimum eigenvalue and the sum of the eigenvalues. Definition of local curvature proposed by Hoppe *et al.* [22] is used in this paper and this method estimates the first order differential of local surface rather than local curvature itself.

Each eigenvalue of the covariance matrix represents the spatial variation along the direction of the corresponding eigenvector. The curvature approximation quantifies the percentage of variance attributed by surface deviation from the tangential plane formed by **e**_1_ and **e**_2_. The ratio of the minimum eigenvalue and the sum of the eigenvalues approximates the curvature, M_curv_(*p*_i_), as follows:

(2) $${\text{M}}_{\text{curv}}(p_{i})=\frac{\lambda_{0}}{\sum_{l=0}^{2}\lambda_{l}}$$

where λ_1_ is the eigenvalue of **e**_1_. Although this method has been demonstrated to provide a good approximation to the change of curvature [2, 28, 5], the quality of estimation depends on how well the neighbourhood points are distributed. The angle between normal vectors and the difference between the changes of curvature of a point and its corresponding points are our criteria for selection of corresponding point pair. Using the information from the previous sections, first the angle between approximate normal vectors of $p_{\text{i}}^{1}$ and $p_{\text{i}}^{2}$ can be expressed as:

(3) $$\theta_{\left(p_{i}^{1};p_{j}^{2}\right)}=\cos^{-1}({\text{n}}_{p_{i}^{1}}\cdot {\text{n}}_{p_{j}^{2}})$$

where ${\text{n}}_{p_{i}^{1}}$ and ${\text{n}}_{p_{j}^{2}}$ are the respective approximate normal vectors of the points. Then the difference in changes of curvature between two points can be written:

(4) $$\beta_{\left(p_{i}^{1};p_{j}^{2}\right)}=\left|M_{\text{cc}}(p_{i}^{1})-M_{\text{cc}}(p_{j}^{2})\right|$$

where $M_{\text{cc}}(p_{i}^{1})$ and $M_{\text{cc}}(p_{j}^{2})$are the approximate changes of curvature of $p_{\text{i}}^{1}$ and $p_{\text{i}}^{2}$. The normal vector of a point is estimated by covariance analysis of the point and its neighbourhood points and the change of curvature is estimated as the ratio of eigenvalues of the covariance matrix.

### 2.2. Description of the proposed algorithm: GP-ICP

The amount of data to process in order to find correspondence is very large, which limits the robustness of registration algorithms. The higher curvature points may have more valuable information than the lower curvature points since they could be edges or corners. Therefore, in the early stages of iteration, we only take into account higher curvature points and then, as iteration proceeds, lower curvature points also are included to improve the registration.

Our method for the registration of three-dimensional, partially overlapping and unorganised point clouds without good a priori alignment can be briefly described as follows. Note that the list of threshold values used in the proposed method is shown in Table 1 and it is assumed that there is no scale different between two point clouds.

1. Find the *k* neighbourhood points of every point in two point clouds named C^1^ and C^2^. Estimate the geometric primitives of the points.
2. Take initial sample points, $p_{\left\{1\ldots n_{\text{iter}=1}\right\}}^{1}$, whose change of curvature is greater than $T_{\text{normal}}^{\text{iter}=i}$ where *n_iter=i_* is the number of sample in the *i*th iteration where $T_{\text{normal}}^{\text{iter}=i}$ is the threshold of the angle between the normal vector in the *i*th iteration. Note that $T_{\text{normal}}^{\text{iter}=i}$ is the threshold values for the difference in the estimated surface normal vectors in the *i*th iteration.
3. Find corresponding points of $p_{\left\{1\ldots n_{\text{iter}=1}\right\}}^{1}$. $p_{j}^{2}$ is the corresponding point of $p_{i}^{1}$ if
  $$\begin{array}{l} \theta_{\left(p_{i}^{1};p_{j}^{2}\right)}\leq T_{\text{normal}}^{\text{iter}=i}\\ \beta_{\left(p_{i}^{1};p_{j}^{2}\right)}\leq T_{\text{cc}}^{\text{iter}=i} \end{array}$$
  where $T_{\text{cc}}^{\text{iter}=i}$ is the threshold for the difference in the changes of geometric curvature between the corresponding points.
4. Calculate the approximate transformation, **Tr***_iter=i_*, and transform C^1^. Rotate the normal vectors of all points of C^1^ as well.
5. Update the threshold values in order to apply a stricter criterion for determination of possible corresponding points as follow.
  $$\begin{array}{l} T_{\text{normal}}^{\text{iter}=i+1}=T_{\text{normal}}^{\text{iter}=i}-\Delta T_{\text{normal}}\\ T_{\text{cc}}^{\text{iter}=i+1}=T_{\text{cc}}^{\text{iter}=i}-\Delta T_{\text{cc}}\\ T_{\text{sample}}^{\text{iter}=i+1}=T_{\text{sample}}^{\text{iter}=i}-\Delta T_{\text{sample}} \end{array}$$
6. Calculate the registration error, ε^iter=i^, which is defined as the rms distance of points and their corresponding surfaces in our method. If ε^iter=i^ is greater than threshold, then go to step Otherwise stop the registration. In addition, if ε^iter=i^ is smaller than *T_εCM_*, for example, the average distance of a point from its neighbourhood, then Chen and Medioni's method is used since it converges quickly than Horn's algorithm does if the point clouds are close. Otherwise Horn's method is used.

If the initial alignment is close to the correct one, only a small number of points need to be searched. Otherwise a large number of points must be searched in order to find correct correspondence of sample points. The optimal number of points being searched could be evaluated from the statistical properties of the distribution of registration error metric [39]. However, the distance distribution of the corresponding points is usually not a unimodal Gaussian but bimodal or multimodal distributions. Furthermore, good initial alignment is not assumed in the proposed method, it is difficult to remove outliers in the early stages of iteration. Therefore, a large number of points need to be searched in order to determine the correspondence of two point clouds.

Among the threshold values utilised in the GP-ICP, $T_{\text{cc}}^{\text{iter}=0}$ and Δ*T*_{_*_normal,cc,sample_*_}_ are the most important and critical thresholds. The other threshold values are not critical to the success rate of the proposed method, although they affect the robustness of the registration. It is difficult to state explicitly which values are the optimal values since they depend on dataset. Currently we are working on finding the optimal and generalized expressions for these thresholds. Our suggestions of $T_{\text{cc}}^{\text{iter}=0}$ and Δ*T*_{_*_normal,cc,sample_*_}_ from the experiences with the proposed method are:

(5) $$T_{\text{cc}}^{\text{iter}=0}=\frac{\left|\langle M_{\text{cc}}^{1}\rangle -\langle M_{\text{cc}}^{2}\rangle \right|}{2}$$

(6) $$\Delta T_{\left\{\text{normal},\text{cc},\text{sample}\right\}}=\sqrt{{\langle M_{\text{cc}}^{1}\rangle }_{\text{rms}}^{2}+{\langle M_{\text{cc}}^{2}\rangle }_{\text{rms}}^{2}}$$

where $\langle M_{\text{cc}}^{i}\rangle$ and ${\langle M_{\text{cc}}^{i}\rangle }_{\text{rms}}$ are the mean and rms of the change of curvature of a point cloud.

## 3. Experimental

### 3.1. Simulated data study

In this section, the precision of the relative transformation parameters by the GP-ICP will be evaluated with a set of simulated point clouds. As mentioned earlier, the accuracy of the estimated parameters can be properly evaluated only with simulated data since the true relative transformation parameters are not available in the other real cases. It must be noted that simulated datasets are unitless. However, in order to help understanding the magnitude of registration or translational errors in a registration algorithm, the pixel of a point cloud is defined as the average distance from a point to its neighbours in the point cloud as follows:

(7) $$\text{pixel}=\frac{1}{n}\sum_{i=1}^{n}\langle D^{pp}(p_{i})\rangle$$

where *n* is the number of points, *k* is the number of neighbours, *D^pp^*(*p_i_*) is the distance between *p_i_* and its *j*th neighbour, and *D^pp^*(*p_i_*) is the average distance between a point and its neighbourhood. Note that the dimension of the pixel of a point cloud is equivalent to that of distance. In addition, the pixel of a three-dimensional point cloud is a relative unit since the size of a pixel is dependent on the spatial characteristics of a point cloud such as point density. However, two partially overlapping point clouds simulated from computer-aided design (CAD) models have almost the same size of a pixel since they have similar spatial point densities. Two simulated point clouds are presented for the GP-ICP convergence region tests: two datasets have been generated from the CAD models, ‘cactus’ and ‘golf club’. These datasets were used in Hoppe *et al.* [22] and are available on ftp://ftp.research.microsoft.com/users/hhoppe/data/thesis/phase2_meshes/. The file names of the cactus and the golf club are cactus.crep1e-5.m.gz and club71.crep1e-5.m.gz, respectively. The points from the CAD models are taken from the three corners of the triangles constituting the CAD models and the cube consists of a set of random points on the surface of a cube.

Two point clouds which share a certain amount of overlapping regions with each other were manually cut from the complete point clouds, e.g. the cactus, the golf club, and the cube. The total number of point clouds and the number of points in the overlapping regions for each point cloud can be found in Table 2.

The convergence region of the GP-ICP will be evaluated in different situations and will be compared with that of the original ICP with random sampling proposed by Besl and McKay [7] and Masuda and Yokoya [26]. The scope of the test for the convergence region of the GP-ICP in this paper with the simulated data must be stated as follows:

- These tests for the convergence region were conducted with the GP-ICP since the convergence region does not heavily depend on whether or not the proposed RANSAC procedure is used, from the fact that the same method for finding correspondence is used in both methods.
- The cactus and the golf club were used for the tests. In all the tests, these simulated point clouds were rotated by a fixed amount, -50° < R*^initial^* < 50°, where R*^initial^* is a rotational angle around an axis. For translations, three kinds of the tests were performed coinciding with the R*^initial^* : no translation, a translation of (H/4, L/4, W/2) named translation 1, and a translation of (-H/4, L/4, W/2) named translation 2, where H, L, and W are the height, length, and the width of either the cactus or the golf club, respectively.
- $T_{\text{normal}}^{\text{iter}=0}$ was set to be R*^initial^* + 10° and it was changed from a maximum value of a test point cloud to zero with increment of 10 pixel. For example, in the test either by the GP-ICP or the original ICP with random sampling, a new $T_{\text{distance}}^{\text{iter}=0}$ is tried unless the solution of a registration algorithm converges, until $T_{\text{distance}}^{\text{iter}=0}$ reaches to zero. Therefore, if the final $T_{\text{distance}}^{\text{iter}=0}$ is zero, it means that the registration algorithm did fail to find a solution within a maximum $T_{\text{distance}}^{\text{iter}=0}$.

**Figure 1.**
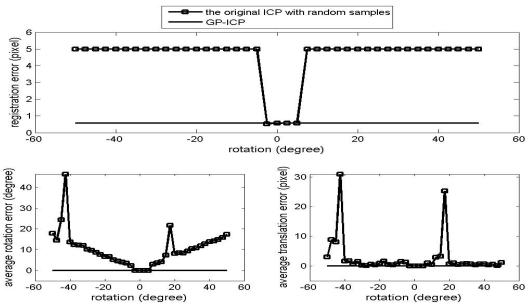
Convergence region test of the point clouds from the cactus without translation.

**Figure 2.**
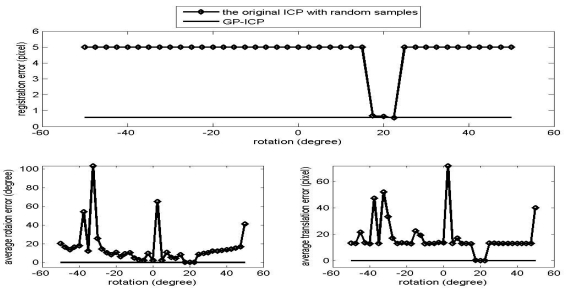
Convergence region test of the point clouds from the cactus with a translation, the translation 1, i.e. (H/4, L/4, W/2).

**Figure 3.**
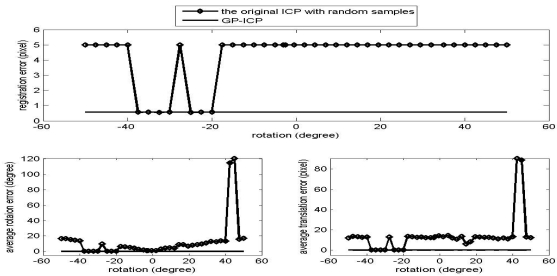
Convergence region test of the point clouds from the cactus with a translation, the translation 2, i.e. (-H/4, L/4, W/2).

**Figure 4.**
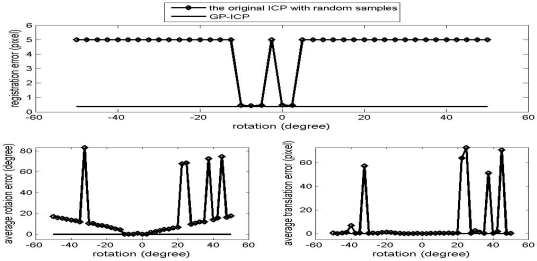
Convergence region test of the point clouds from the gold club without translation.

**Figure 5.**
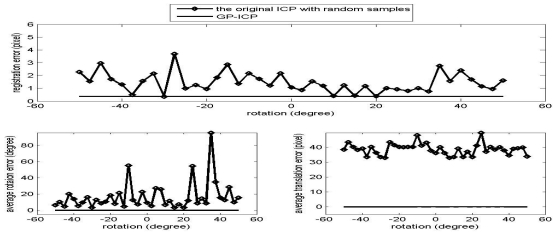
Convergence region test of the point clouds from the golf club with a translation, the translation 2, i.e. (H/4, L/4, W/2).

**Figure 6.**
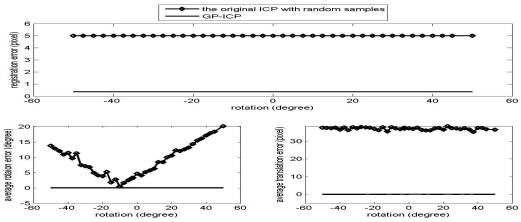
Convergence region test of the point clouds from the golf club with a translation, the translation 1, i.e. (-H/4, L/4, W/2).

The results of the convergence tests of both the GP-ICP and the original ICP with random sampling are presented in Figures 1 – 6. In all the tests, the GP-ICP provides ±50° rotational convergence region, which is the maximum possible rotational convergence angle in these tests. On the other hand, the original ICP with random sampling's rotational convergence region in these tests is at best ±50°. For example, in the case of Figures 5 and 6, the original ICP with random sampling does not converge into a solution in any test region, i.e. the rotational convergence region is zero. Although the GP-ICP requires a different $T_{\text{distance}}^{\text{iter}=0}$ in a different situation, the convergence region of the GP-ICP is reasonably large for practical applications.

The success rate of the original ICP with random samples is poor since it does not escape from local minima in the ways that it finds a corresponding point, i.e. using the nearest neighbour point as the corresponding point. The GP-ICP provides a way of avoiding these kinds of local minima, although it still has limitations. In the case of the direct georeferencing method, the translation parameters usually converge more easily. In other words, finding possible corresponding points is a part of the problem for the GP-ICP, the ICP, or its variants. In these cases, finding the translational parameters is more difficult than the rotational parameters since the estimated translational parameters are simply the translational differences in the centroids of the selected corresponding points, unless a weighted least square method is employed. In the case of direct georeferencing methods, to find the translational parameters is easier, only because a good set of corresponding points is already given.

**Figure 7.**
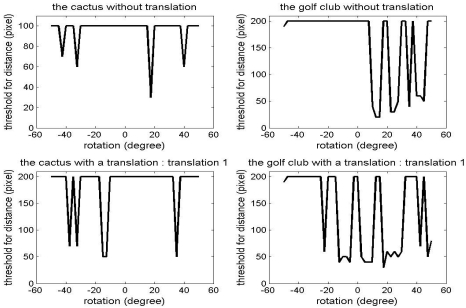
$T_{\text{distance}}^{\text{iter}=0}$ for the convergence region tests in Section 3.1.

Figure 7 shows the initial threshold for distance, $T_{\text{distance}}^{\text{iter}=0}$, in the convergence tests performed in the previous section. As explained earlier, $T_{\text{distance}}^{\text{iter}=0}$ was decreased from a maximum value to zero in these tests. If a solution close to the truth is found, then the iteration was stopped and the current $T_{\text{distance}}^{\text{iter}=0}$ was recorded. For example, in the case of the cactus without translation, i.e. Figure 7(a), the maximum $T_{\text{distance}}^{\text{iter}=0}$ is set to 100 pixels. Basically, we would like to have a constant $T_{\text{distance}}^{\text{iter}=0}$ over the entire convergence region of a registration method. However, in the cases of the simulated data, the required $T_{\text{distance}}^{\text{iter}=0}$ with which a good estimation of the true relative transformation of the point clouds is obtained, changes in an unpredictable manner mainly because its point density is much lower than that of either close-range or terrestrial laser scanner data. The probability of finding a good set of corresponding points is decreased with a larger $T_{\text{distance}}^{\text{iter}=0}$. Fortunately, it will be shown in later sections that a similar level of the rotational convergence region with the simulated datasets is maintained with the tests with point clouds on both close-range and terrestrial laser scanners with a smaller deviation in $T_{\text{distance}}^{\text{iter}=0}$.

### 3.2. Real case study with close-range laser scanner

**Figure 8.**
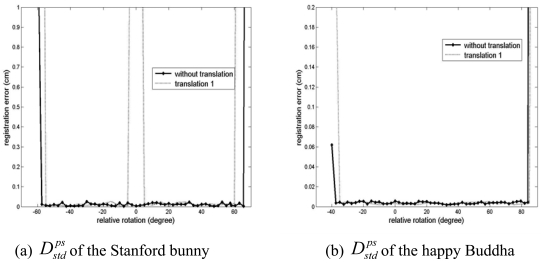
Convergence region tests of datasets from the Stanford 3D repository for the GP-ICP. The zero rotations of the curves represent different relative transformations of the data. (a) and (b) are the $D_{\text{std}}^{\text{ps}}$ of the registered point clouds: the Stanford bunny and the happy Buddha, respectively.

**Figure 9.**
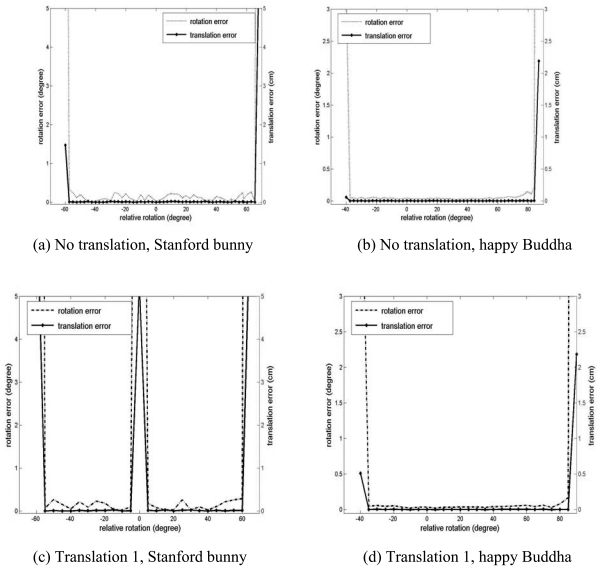
Registration errors of data from the Stanford 3D repository using the GP-ICP. (a) and (b) are the errors in the estimated transformation parameters by the GP-ICP for the Stanford bunny and the happy Buddha, respectively, without a relative translation. (c) and (d) are the same values when a point cloud is translated to translation 1 of the datasets.

For tests of the precision of the GP-ICP, two datasets from the Stanford 3D scanning repository will be used: the “Stanford bunny” and the “happy Buddha” as these are referred to by computer graphics researchers. These datasets were obtained from the Stanford 3D scanning repository [35] which were scanned with a Cyberware 3030MS [13]. These datasets have approximately ten point clouds and two sets of them from both the Stanford bunny and the happy Buddha will be used in this section. One point cloud of the Stanford bunny was named ‘bunny000’ following its original file name, bun000.ply. The other was named ‘bunny090’, again following its original file name, bun090.ply. One point cloud of the happy Buddha was named ‘happybuddha_StandRight_0’ after its original file name, happyStandRight_0.ply. The other was named ‘happybuddha_StandRight_48’ again after its original file name, happyStandRight_48.ply. It must be noted that the pre-processed data from one of the Stanford graphics research group's smoothing procedures, e.g. Curless and Levoy [12], were not used in this thesis. Instead, a set of raw point clouds from the close-range scanner was utilised to evaluate the performance of GP-ICP. In addition, the pixel sizes of the Stanford bunny and the happy Buddha are about 2.30mm and 1.14mm, respectively. The convergence region of the GP-ICP will be evaluated using close-range laser scanner datasets, i.e. the Stanford bunny and the happy Buddha. The scope of the tests can be stated as follows:

- Unlike the convergence region tests with the simulated data, the Stanford bunny and the happy Buddha were, from the registered state, rotated around an axis in both clockwise and counter-clockwise until the GP-ICP fails to obtain a solution. Therefore, a point cloud's rotational convergence region can be asymmetric, e.g. -40° < R*^initial^* < 20°, where R*^initial^* is the rotational convergence region of a point cloud.
- Since the true transformation was known and this test was designed to evaluate the convergence region of the GP-ICP, the algorithm was stopped if the difference between the true and the estimated transformation parameters was sufficiently small, regardless of the magnitude of the registration error in the last iteration. In other words, in this test, the GP-ICP did not try to find the smallest possible registration error. Therefore, in the plot of $D_{\text{std}}^{\text{ps}}$ in Figure 8, a little fluctuation is observed in the registration errors. In addition, a similar fluctuation is observed in the errors of the estimated transformation parameters as shown in Figure 9.
- $T_{\text{normal}}^{\text{iter}=0}$ was again set to be R*^initial^* + 10°. A threshold for distance, $T_{\text{distance}}^{\text{iter}=0}$, was changed from a maximum value to zero with increment of 5cm. As stated in Section 3.1, if the final $T_{\text{distance}}^{\text{iter}=0}$ is zero, it means that a registration algorithm failed to find a good solution.

The results of the convergence test of the GP-ICP with the two Stanford datasets are presented in Figure 8. In the case of no translation, the rotational convergence region of two datasets from the happy Buddha is about -50° < R*^initial^* < 60° and that of two datasets from the Stanford bunny is about -40° < R*^initial^* < 80°. As the happy Buddha was translated by (H/4, L/4, W/2) which was named ‘translation 1’ of the object in Section 3.1, we have almost the same rotational convergence region as the cases without translation of the happy Buddha. However, in the case of the Stanford bunny translated by its translation 1, there is a region of discontinuity in the rotational convergence region, about ±5° from its translation 1 as shown in Figures 8(a) and 9(c).

This is a kind of slide effect mentioned by Rusinkiewicz and Levoy [32]. In their cases, the slide effect refers to the case in which it is difficult to find a set of corresponding points when there are only a small number of geometrically distinguishable features, e.g. a point cloud of an engraved plate. This can also be explained in terms of the method of collecting samples for finding a set of possible corresponding points. In GP-ICP, we first select a set of the nearest neighbours in the point cloud of a query point from the other point cloud. Then the best possible corresponding point for the query point is selected as explained in Section 2.

**Figure 10.**
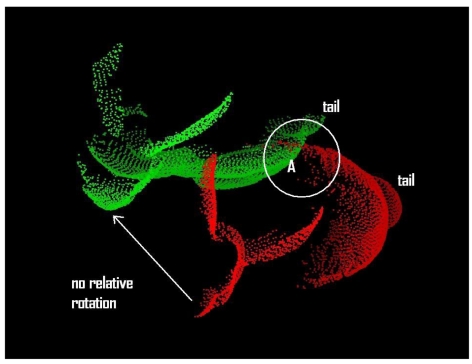
Bunny000 and bunny090 translated to translation 1 with zero relative rotation. There is no relative rotation and the relative translation is (H/4, L/4, W/2) where H, L, and W are the height, length, and width of the object, i.e. the Stanford bunny.

For example, the nearest red neighbours of a green query point in the region A of Figure 10 are not the corresponding points of the green query point. Its true corresponding point is far from region A. In many cases, this problem is avoided using GP-ICP for finding corresponding points as observed in the convergence test of the happy Buddha in the cases of either with or without translation. This problem exists in the Stanford bunny but not in the happy Buddha since the happy Buddha has more geometrically distinct features, i.e. higher curvature points, than the Stanford bunny.

**Figure 11.**
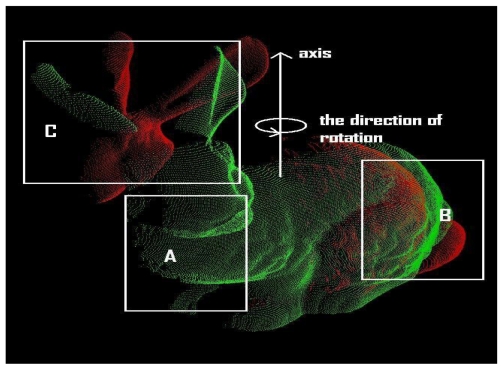
Schematic outline of the convergence tests with the Stanford bunny. The axis and the direction of the initial rotations for the convergence tests are presented. The red point cloud, the bunny090, is rotated around the axis either to or against the direction of rotation in the picture.

The asymmetry in the rotational convergence region is mainly caused by the ratio of overlapping regions and the geometric shape of an object. It is also observed that the asymmetry in the Stanford bunny is relatively smaller than that of the happy Buddha. Figure 11 is utilised to explain this asymmetry in the rotational convergence region. The Stanford bunny is chosen for the explanation of asymmetry in the rotational convergence region since it is much more visually clearer and easier to explain than it is for the happy Buddha. The positive rotation is indicated by the direction of rotation in Figure 11.

It can be clearly seen that there are almost no corresponding points in region A between bunny000 and bunny090. Furthermore, region B has a larger set of corresponding points between the point clouds than region C. In addition, in region B we find many more distinctive regions than in region C, in terms of the geometric shape of the regions. Although the parts of the Stanford bunny's ears are in region C, the change of curvature around region C is much lower than that of region B. The presence of a higher curvature region around the ears is not much help for finding a set of corresponding points. Simply speaking, with either the GP-ICP or a modified ICP algorithm based on geometric roughness, the algorithm has a much larger probability of finding a set of possible corresponding points in region B than in region C. That is why there is approximately 60° rotational convergence region in the positive direction of rotation. In the negative direction of rotation, there is a 50° rotational convergence region. In general, for this test of the convergence region of the GP-ICP, we have at least ±40° rotation convergence region with two point clouds from either the Stanford bunny or the happy Buddha. This rotational convergence region is large enough for practical applications using either terrestrial or close-range laser scanners.

**Figure 12.**
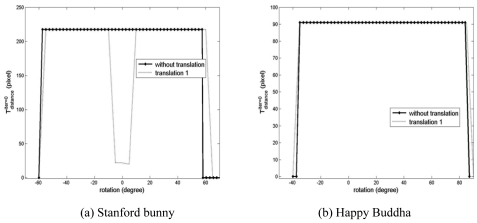
Initial thresholds for distance, i.e., $T_{\text{normal}}^{\text{iter}=0}$ for the registration tests of the Stanford bunny and the happy Buddha using the GP-ICP without translation.

As shown in the results of Section 3.1, the required $T_{\text{distance}}^{\text{iter}=0}$ with which a good estimation of the true relative transformation of the point clouds is obtained, changed in an unpredictable manner in the convergence region test with the simulated datasets. In the case of close-range laser scanner data, the GP-ICP provides a precise estimation of the relative transformation of the point clouds with a steadier and smaller deviation in $T_{\text{distance}}^{\text{iter}=0}$ as presented in Figure 12.

### 3.3. Real case study with terrestrial laser scanner

The convergence region of the GP-ICP with terrestrial laser scanner data will be evaluated in this section. This dataset and surveying information (Agia Sanmarina church in Greece) is currently available through ISPRS WG V/3 Terrestrial Laser Scanning (http://www.commission5.isprs.org/wg3), which was acquired from nine different locations around the church. The point clouds were named after the locations of the laser scanner, e.g. east or southeast. The number of points for Figures 15(a) and 15(b) is about 50,000. The radial distance between the church and the scanner is approximately 20 m. The dimension of the church is approximately (L, W, H) = (25.0 m, 15.0 m, 10.0 m) where L, W, and H are the length, width, and height of the object, respectively.

A rotational convergence region of a set of point clouds with a registration algorithm is dependent on both its relative translation and $T_{\text{distance}}^{\text{iter}=0}$. The convergence region of terrestrial laser scanner data is especially dependent on $T_{\text{distance}}^{\text{iter}=0}$ since a terrestrial laser scanner measures physically larger objects than a close-range laser scanner. In other words, the point clouds from terrestrial laser scanners usually require a much larger $T_{\text{distance}}^{\text{iter}=0}$ for the same relative rotation angle than close-range scanner datasets. Therefore, it is more difficult to properly find the convergence region of a registration method. Note that $T_{\text{distance}}^{\text{iter}=0}$ is again changed from a maximum value of a test point cloud to zero with an increment of 5 cm. If a required $T_{\text{distance}}^{\text{iter}=0}$ is changed greater than the current test $T_{\text{distance}}^{\text{iter}=0}$, then the required $T_{\text{distance}}^{\text{iter}=0}$ can never be found unless manually changed, i.e. to set a new and larger maximum value. Therefore, the results in this section must be regarded as the rotational convergence region for terrestrial laser scanners with a fixed maximum $T_{\text{distance}}^{\text{iter}=0}$. In fact, this has been true for all the convergence region tests in this paper.

**Figure 13.**
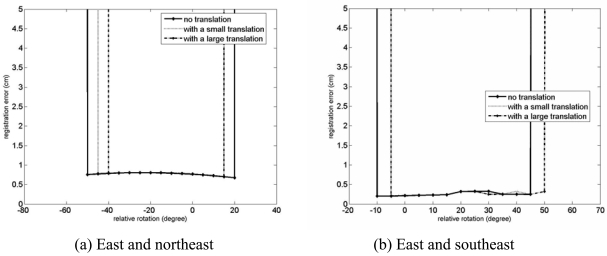
Registration errors, $D_{\text{std}}^{\text{ps}}$, for the convergence region test of the GP-ICP with the Agia Sanmarina church data. Large and small translations are [dx, dy, dz] = [0.5 m, 0.5 m, 1.0 m] and [0.1 m, 0.1 m, 0.1 m], respectively. The northeast of the Agia Sanmarina church was rotated around the y axis which is about the surface normal of the ground.

**Figure 14.**
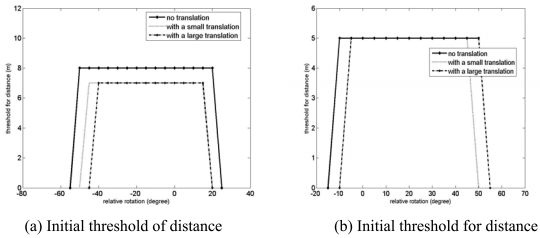
Initial threshold for distance, $T_{\text{distance}}^{\text{iter}=0}$, for the convergence region test of the GP-ICP with the Agia Sanmarina church data. A large and small translations are [dx, dy, dz] = [0.5 m, 0.5 m, 1.0 m] and [0.1 m, 0.1 m, 0.1 m], respectively. The northeast of the Agia Sanmarina church was rotated around the y axis which is approximately the surface normal of the ground.

**Figure 15.**
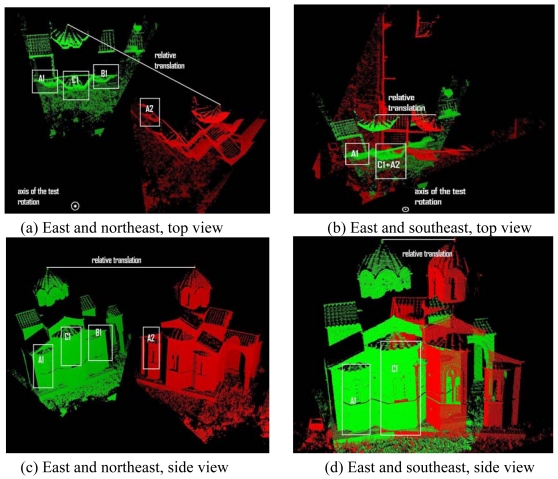
Top and side views of the Agia Sanmarina church data for the convergence region test in the case of the large translation, i.e. [dx, dy, dz] = [0.5 m, 0.5 m, 1.0 m]. Green is the east of the church. The red point clouds in (a) and (b) are the northeast and the southeast of the church, respectively.

The results of the convergence test are presented in Figures 13 and 14. Note that the positive direction of the rotation around the y axis is counter-clockwise around the axis of the test rotation as shown in Figure 15. For the case of the registration of the east and the northeast clouds, the rotation convergence region is about -50° < R*^initial^* < 20° with no relative translation.In addition, in presence of the relative translation, it is observed that the rotational convergence region is reduced and shifted to the positive direction of the rotation by an amount in the order of 5°. In the case of the registration of the east and the southeast clouds, about -10° < R*^initial^* < 45° to be the rotational convergence region is observed, with no relative translation. In the presence of relative translations, the shift of the rotational convergence region is also observed, although the absolute size of the rotational convergence region is about the same as in the case of no relative translation.

Figure 14 shows that a very rough estimation of the required $T_{\text{distance}}^{\text{iter}=0}$ is only required for the proper registration of a set of point clouds. For example, as shown in Figure 14(a), 8m and 7m were used for $T_{\text{distance}}^{\text{iter}=0}$ over the entire convergence region in cases both with and without relative translation for the registration of the east and the northeast point clouds. Even if the absolute size of the rotation convergence region of the registration of terrestrial laser scanner data is large enough for practical applications, an asymmetry in the rotational convergence region is still observed as seen the cases for close-range laser scanner data.

In order to explain the cause of this asymmetry in the rotational convergence region, the top and side views of the parts of the Agia Sanmarina church data are presented in Figure 15. Note that region A1 in the green is the corresponding region of A2 in the red. In Figures 15(a) and 15(c), the northeast of the church is rotated by -50° around the y axis, i.e. the axis of the test rotation, and also translated [dx, dy, dz] = [0.5 m, 0.5 m, 1.0 m], i.e. the large translation in Figure 13. This transformation is one of the limits of the convergence region as shown in Figure 13(a). It is observed that region B1 of the church is the closer region to region A2 than its true corresponding region, i.e. A1. In addition, the geometric shape of region B1 is very similar with region A1 and furthermore to reach region A1, we need to go through region C1, which has a similar shape but a different scale. In the case of the east and the southeast clouds presented in Figures 15(b) and 15(d), the latter is rotated around y axis by -15° and again translated by [0.5 m, 0.5 m, 1.0 m]. Note that this transformation is also a limit of the convergence region between the east and the southeast of the church as shown in Figure 13(b). In this case, a smaller rotation convergence limit is achieved with the same translation because region A2 is much closer to region C1 than A1. Therefore, the GP-ICP is likely to find a possible corresponding set of region A2 in region C1 rather than A1.

## 4. Conclusions

These tests on the convergence region of the GP-ICP effectively demonstrate that the proposed method has about 1m translational and on the order of 10° rotational convergence with terrestrial laser scanner datasets. Although there is room for improvement to achieve a fully automated registration of three-dimensional point clouds, the current level of rotational and translational convergence region of the GP-ICP was demonstrated to be effect for on-site registration of point clouds from a terrestrial laser scanner.

## Figures and Tables

**Table 1.** Threshold values are used in the GP-ICP.

**Threshold**	**Description**
*k*	Number of neighbourhood
$T_{\text{sample}}^{\text{iter}=0}$	Initial sampling threshold for the change of curvature
$T_{\text{sample}}^{\text{iter}=0}$	Initial threshold for the difference in charge of curvature
$T_{\text{sample}}^{\text{iter}=0}$	Initial threshold for the angle between normal vectors
Δ*T*_{_*_sample,cc,normal_*_}_	Increment for Δ*T*_{_*_sample,cc,normal_*_}_
Δ*T_εCM_*	Threshold for starting Chen and Medioni's method
*T_ε_*	Threshold for stopping the algorithm

**Table 2.** Sizes of the overlapping regions are represented as percentages of the whole point cloud, i.e. the denominator is the total number of points in the point cloud and the numerator is the number of points in the overlapping region.

	**Point cloud 1**	**Point cloud 2**
**Cactus**	179/519 = 34%	179/545 = 33%
**Gold club**	622/1371 = 45%	622/1861 = 33%

## References

[1] Bae K.-H. (2006). Automated registration of unorganised point clouds from terrestrial laser scanners. PhD thesis.

[2] Bae K.-H., Lichti D.D. (2004). Automated registration of unorganised point clouds from terrestrial laser scanners. Int. Arch. Photogram. Rem. Sens..

[3] Bae K.-H., Lichti D.D. (2008). A method for automated registration of unorganised point clouds. ISPRS J. Photogramm. Remote Sens..

[4] Bae K.-H., Belton D., Lichti D.D. (2005). A framework for position uncertainty of unorganized three-dimensional point clouds from near-monostatic laser scanners using covariance analysis. Int. Arch. Photogram. Rem. Sens. Spatial Inform. Sci..

[5] Bae K.-H., Belton D., Lichti D.D. (2007). Pre-processing procedures for raw point clouds from terrestrial laser scanners. J. Spatial Sci..

[6] Barnea S., Filin S. (2008). Keypoint based autonomous registration of terrestrial laser point clouds. ISPRS J. Photogram. Rem. Sens..

[7] Besl P.J., McKay N.D. (1992). A method for registration of 3-D shapes. IEEE Trans. Pattern Anal. Mach. Intell..

[8] Brenner C., Dold C., Ripperda N. (2008). Coarse orietation of terrestrial laser scans in urban environments. ISPRS J. Photogram. Rem. Sens.

[9] Campbell R.J., Flynn P.J. (2001). A survey of free-form object representation and recognition techniques. Comput. Vision Image Understand..

[10] Chen Y., Medioni G. (1992). Object modelling by registration of multiple range images. Image Vision Comput..

[11] Chua C.S., Jarvis R. (1996). 3D free-form surface registration and object recognition. Int. J. Comput. Vision..

[12] Curless B., Levoy M. (1996). A volumetric method for building complex models from range images.

[13] Cyberware Homepage.

[14] Dold C. (2005). Extended Gaussian images for the registration of terrestrial scan data. Int. Arch. Photogram. Rem. Sens. Spatial Inform..

[15] Dyn N., Hormann K., Kim S.-J., Levin D. (2001). Optimizing 3D triangulations using discrete curvature analysis.

[16] Fitzgibbon A. (2003). Robust registration of 2D and 3D point sets. Image Vision Comput..

[17] Frank T., Tertois A.-L., Mallet J.-L. (2007). 3D-reconstruction of complex geological interfaces from irregularly distributed and noisy point data. Comput. Geosci..

[18] Golub G.H., Loan C.F.V. (1989). Matrix computations.

[19] Gruen A., Akca D. (2005). Least squares 3D surface and curve matching. ISPRS J. Photogram. Rem. Sens..

[20] Haralick R.M., Joo H., Lee C.H., Zhang X., Vaidya V.G., Kim M.B. (1989). Pose estimation from corresponding point data. IEEE Trans. Syst. Man Cybern..

[21] Higuchi K., Hebert M., Ikeuchi K. (1995). Building 3-D models from unregistered range images. Graph. models image Process..

[22] Hoppe H., DeRose T., Duchamp T., McDonald J., Stuetzle W. (1992). Surface reconstruction from unorganized points. Comput. Graphics..

[23] Horn B.K.P. (1984). Extended Gaussian images. Proc. IEEE.

[24] Horn B.K.P. (1987). Closed-form solution of absolute orientation using unit quaternions. J. Opt. Soc. Am..

[25] Johnson A.E., Hebert M. (1999). Using spin images for efficient object recognition in cluttered 3D scenes. IEEE Trans. Pattern Anal. Mach. Intell..

[26] Masuda T., Yokoya N. (1995). A robust method for registration and segmentation of multiple range images. Comput. Vision Image Understand..

[27] Miliaresis G., Kokkas N. (2007). Segmentation and object-based classification for the extraction of the building class from LIDAR DEMs. Comput. Geosci..

[28] Pauly M., Mitra N.J., Guibas L.J. (2004). Uncertainty and variability in point cloud surface data.

[29] Pottmann H., Huang Q.-X., Yang Y.-L., Hu S.-M. (2006). Geometry and convergence analysis of algorithms for registration of 3d shapes. Int. J. Comput. Vision..

[30] Rabbani T., Dijkman S., van den Heuvel F., Vosselman G. (2007). An integrated approach for modelling and global registration of point clouds. ISPRS J. Photogram. Rem. Sens..

[31] Rodrigues M., Fisher R., Liu Y. (2002). Special issue on registration and fusion of range images. Comput. Vision Image Understand..

[32] Rusinkiewicz S., Levoy M. (2001). Efficient variant of the ICP algorithm.

[33] Sharkarji C.M. (1998). Least-squares fitting algorithms of the NIST algorithm testing system. J. Res. Natl. Inst. Stand. Technol..

[34] Sharp G.C., Lee S.W., Wehe D.K. (2002). ICP registration using invariant features. IEEE Trans. Pattern Anal. Mach. Intell..

[35] The Stanford 3D Scanning Repository.

[36] Suárez J.C., Ontiveros C., Smith S., Snape S. (2005). Use of airborne LiDAR and aerial photogrammetry in the estimation of individual tree heights in forestry. Comput. Geosci..

[37] Tang C.-K., Medioni G. (2002). Curvature-augmented tensor voting for shape inference from noisy 3d data. IEEE Trans. Pattern Anal. Mach. Intell..

[38] Umeyama S. (1991). Least squares estimation of transformation parameters between two patterns. IEEE Trans. Pattern Anal. Mach. Intell..

[39] Zhang Z. (1994). Iterative point matching for registration of free-form curves and surfaces. Int. J. Comput. Vision.

